# First Reported Case of Dulaglutide-Induced Acute Pancreatitis With Normal Serum Lipase Level

**DOI:** 10.7759/cureus.40576

**Published:** 2023-06-17

**Authors:** Mohammad Shahbazi, Zainab Qudsiya, Aboud Fahel, Afshin Amini, Tariq Tanoli

**Affiliations:** 1 Internal Medicine, St. Luke's Hospital, Chesterfield, USA; 2 Endocrinology, Diabetes and Metabolism, St. Luke's Hospital, Chesterfield, USA

**Keywords:** victoza, ozempic, trulicity, exenatide, liraglutide or saxenda, semaglutide, glp-1 agonist, normal lipase pancreatitis, dulaglutide, acute pancreatitis (ap)

## Abstract

Dulaglutide is being extensively used for non-insulin-dependent diabetes mellitus and congestive heart failure and is also being used as an off-label weight loss aid. Due to its wide use, we had to shed some light on this rare finding of normal lipase level in a patient with signs and symptoms suggestive of acute pancreatitis. A high index of clinical suspicion for acute pancreatitis despite normal lipase should warrant a low threshold for radiological imaging to rule it out.

## Introduction

Acute pancreatitis is an inflammatory condition of the pancreas presenting with abdominal pain and elevation of pancreatic enzymes in the blood. Acute pancreatitis is a leading gastrointestinal cause of hospital admissions in the United States [1]. Gallstones and alcohol are the established leading factors of acute pancreatitis. Many drugs are known to induce acute pancreatitis and occur with serum lipase elevation [2]. Here, we report a rare case of dulaglutide-induced acute pancreatitis (AP) with normal serum lipase that has not been previously reported, to the best of our knowledge.

## Case presentation

**Figure 1:**
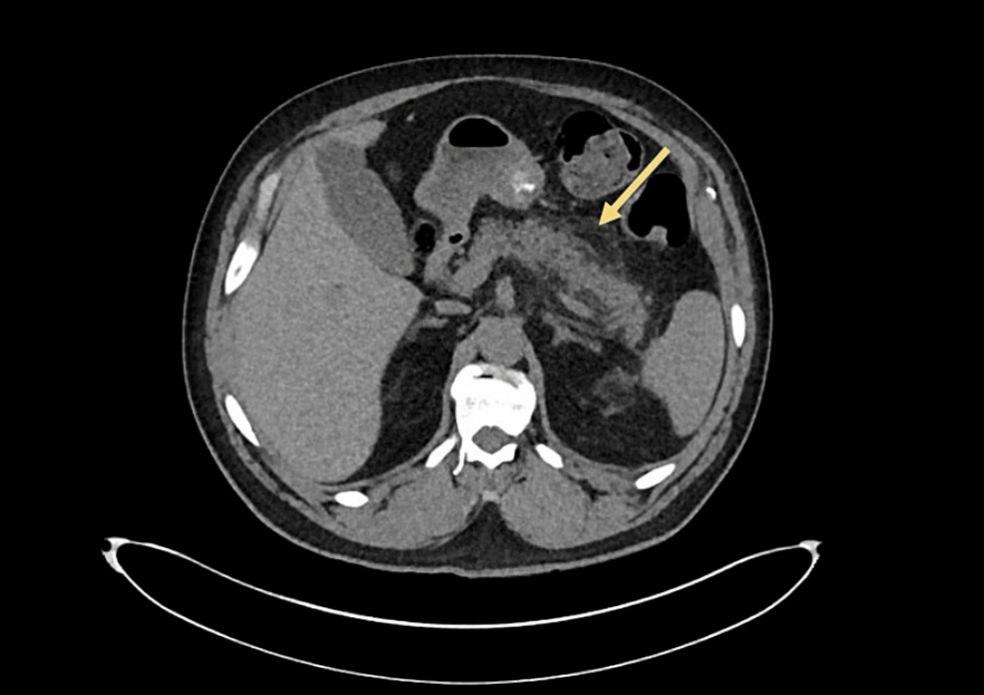
CT abdomen/pelvis w/o contrast showing extensive interstitial edema around the pancreatic tail along with peripancreatic fat stranding

A 56-year-old Caucasian man, with a history of heart failure, chronic obstructive pulmonary disease (COPD), type 2 diabetes mellitus (DM), and acute lymphocytic leukemia in remission, presented with diffuse abdominal pain and progressive constipation for four weeks. Dulaglutide was initiated four weeks prior to presentation with a recent dose increase from 0.75 mg to 1.5 mg. After up-titration, he reported dose-related nausea, abdominal pain, and a decline in bowel movement frequency from his baseline of once every day to once every three days. He denied alcohol intake, smoking, or other medication changes. On exam, he was afebrile, hemodynamically stable, with mild epigastric tenderness. Investigations including WBC (10,600/uL), serum lipase (171 U/L), bilirubin, alkaline phosphatase (ALP), and triglycerides were normal. CT abdomen with contrast revealed acute pancreatitis with extensive interstitial edema, fat stranding along the pancreatic tail, and a moderate amount of colonic stool (Figure 1). The patient was admitted with acute pancreatitis secondary to dulaglutide use, which was discontinued, kept nil by mouth, and given IV hydration, rectal bisacodyl, and linaclotide. His abdominal pain gradually improved, he had multiple bowel movements, and his diet was advanced. He was discharged home in three days with instructions to follow up with his endocrinologist.

## Discussion

Acute pancreatitis with a normal serum lipase level is rare, with only a few cases reported worldwide with varying suspected triggers [3,4]. The diagnosis of AP requires two of three criteria: 1) serum lipase/amylase elevation three times or greater than the upper limit of normal; 2) upper abdominal pain radiating to the back; 3) characteristic radiographic findings of AP on imaging (presence of focal or diffuse enlargement of the pancreas on contrast-enhanced abdominal CT or MRI) [2]. Serum amylase and lipase are often considered important laboratory tools to diagnose acute pancreatitis. Intra-acinar activation of pancreatic proteolytic enzymes is one of the earliest pathogenic events in acute pancreatitis that lead to pancreatic autodigestion and subsequent leakage of these enzymes into the systemic circulation. Serum lipase measurement is preferred above amylase as levels rise earlier (4-8 hours vs. 6- 13 hours), with prolonged elevation (8-14 days), high negative predictive value, and organ specificity. This makes lipase a valuable tool in diagnosing late presentations and excluding acute pancreatitis [5,6]. Further, due to its high negative predictive value (94-100%), lipase is a valuable diagnostic tool to exclude acute pancreatitis [7]. Due to these characteristics, the Choosing Wisely campaign recommends the measurement of lipase rather than amylase or co-testing as a diagnostic tool for acute pancreatitis [8].

To our knowledge, this is the first reported case of dulaglutide-induced pancreatitis with a normal lipase level. Limon et al. reported two cases of acute pancreatitis, one of which was associated with triglyceride levels (806 mg/dL) above the normal limit but below the threshold commonly associated to cause acute pancreatitis (1000 mg/dL) [9]. Mathur et al. reported acute pancreatitis with normal lipase in two patients with a history of heavy alcohol use [10]. Tauseef et al. reported a case of inflammatory bowel disease associated with acute pancreatitis and normal lipase with atypical lower abdominal pain [11]. A case series by Shah et al. of eight patients with normal lipase acute pancreatitis identified possible triggers as DM in two patients, gallstones in two patients, and alcohol in one patient [12]. There is a paucity of data on drug-induced AP with normal lipase. Karkee et al. reported a case of a 38-year-old female with migraine with suspected sumatriptan-induced acute pancreatitis with normal lipase [7].

GLP1 receptor agonists have recently emerged at the forefront for type 2 diabetes mellitus and weight loss management and have attracted attention due to their possible association with acute pancreatitis. Although results from meta-analysis and systematic reviews indicate a low overall risk (0.1%) of pancreatitis, all GLP1 agonists carry warnings for acute pancreatitis [13]. Exenatide, one of the first GLP1 agonists to be introduced, had 36 post-marketing reports of acute pancreatitis and was associated with a six-fold higher risk of pancreatitis compared to other diabetic medications in the first two years of use [14,15]. Similarly, other GLP1A have elevated risk of acute pancreatitis: liraglutide (1.6 cases/1000 patient-years) and dulaglutide (1.4 cases/1,000 patient-years vs 0.88 cases/1,000 patient-years compared to non-incretin therapy) [16,17]. Proposed mechanisms of GLP-1 agonist-induced pancreatitis include pancreatic duct gland hyperplasia, acinar cell hypertrophy, pancreatic ductule obstruction leading to proinflammatory reaction, and pancreatic vascular injury [14,17]. GLP1 agonists have also been noted to cause subclinical pancreatic inflammation and pancreatic enzyme elevation within the normal limits, the significance of which is unknown. In regard to our case, it is possible that the delayed presentation contributed to normal lipase levels. While pancreatic enzyme elevation is associated with the GLP-1 agonist, there are no reports of GLP-1 agonist-induced acute pancreatitis with normal lipase levels as seen in this case.

## Conclusions

This case emphasizes the importance of monitoring patients on GLP1A for new-onset gastrointestinal complications including acute pancreatitis that may have an atypical presentation. Clinicians should avoid solely relying on serum lipase and maintain a low threshold for abdominal imaging in such patients to avoid missing a diagnosis of acute pancreatitis that requires prompt discontinuation of the drug.
